# Suppression of exaggerated NMDAR activity by memantine treatment ameliorates neurological and behavioral deficits in aminopeptidase P1-deficient mice

**DOI:** 10.1038/s12276-022-00818-9

**Published:** 2022-08-03

**Authors:** Young-Soo Bae, Sang Ho Yoon, Young Sook Kim, Sung Pyo Oh, Woo Seok Song, Jin Hee Cha, Myoung-Hwan Kim

**Affiliations:** 1 Department of Physiology and Biomedical Sciences, Seoul National University College of Medicine, Seoul, 03080 Korea; 2 Neuroscience Research Institute, Seoul National University Medical Research Center, Seoul, 03080 Korea; 3 Seoul National University Bundang Hospital, Seongnam, Gyeonggi 13620 Korea

**Keywords:** Cellular neuroscience, Developmental disorders

## Abstract

Inborn errors of metabolism (IEMs) are common causes of neurodevelopmental disorders, including microcephaly, hyperactivity, and intellectual disability. However, the synaptic mechanisms of and pharmacological interventions for the neurological complications of most IEMs are unclear. Here, we report that metabolic dysfunction perturbs neuronal NMDA receptor (NMDAR) homeostasis and that the restoration of NMDAR signaling ameliorates neurodevelopmental and cognitive deficits in IEM model mice that lack aminopeptidase P1. Aminopeptidase P1-deficient (Xpnpep1^–/–^) mice, with a disruption of the proline-specific metalloprotease gene *Xpnpep1*, exhibit hippocampal neurodegeneration, behavioral hyperactivity, and impaired hippocampus-dependent learning. In this study, we found that GluN1 and GluN2A expression, NMDAR activity, and the NMDAR-dependent long-term potentiation (LTP) of excitatory synaptic transmission were markedly enhanced in the hippocampi of Xpnpep1^–/–^ mice. The exaggerated NMDAR activity and NMDAR-dependent LTP were reversed by the NMDAR antagonist memantine. A single administration of memantine reversed hyperactivity in adult Xpnpep1^–/–^ mice without improving learning and memory. Furthermore, chronic administration of memantine ameliorated hippocampal neurodegeneration, hyperactivity, and impaired learning and memory in Xpnpep1^–/–^ mice. In addition, abnormally enhanced NMDAR-dependent LTP and NMDAR downstream signaling in the hippocampi of Xpnpep1^–/–^ mice were reversed by chronic memantine treatment. These results suggest that the metabolic dysfunction caused by aminopeptidase P1 deficiency leads to synaptic dysfunction with excessive NMDAR activity, and the restoration of synaptic function may be a potential therapeutic strategy for the treatment of neurological complications related to IEMs.

## Inherited neurodevelopmental disorders: Repairing neural circuitry as a treatment

Addressing neurological symptoms may offer new treatments for inborn errors of metabolism (IEMs) affecting neurodevelopment. In such IEMs, mutation of an enzyme disrupts a metabolic pathway, causing buildup or lack of key molecules, with symptoms including hyperactivity, developmental delay, and intellectual disability. Because the detailed pathological mechanisms of most IEMs are unknown, there are no treatments for resulting neurological issues. Myoung-Hwan Kim at Seoul National University and co-workers investigated whether they could treat the neurological symptoms of the IEM, aminopeptidase P1 (APP1) deficiency. They found that APP1 deficiency in mice caused an increase in the neural receptor NMDAR. Suppressing NMDAR reduced both neurological and behavioral symptoms. These findings suggest potential treatments for APP1 deficiency, and indicate that neurodevelopmental disorders in IEMs may be treated by repairing the neural circuitry instead of the root metabolic cause.

## Introduction

Inborn errors of metabolism (IEMs), also known as inheritable metabolic diseases, are caused mainly by mutations in a single gene that encodes an enzyme in a specific metabolic pathway^1^. Although an individual IEM is rare (incidence < 1:100,000) owing largely to a recessive inheritance pattern, IEMs are collectively common disorders with an incidence of 1:800–2500 births and account for more than 15% of single-gene disorders^2–4^. To date, more than 1000 distinct IEMs have been identified^5^, and the most serious and common outcomes in IEMs are neurodevelopmental disorders, such as developmental delay, microcephaly, hyperactivity, attention deficit, autism, and intellectual disability^6–11^. The neural circuit mechanisms underlying neurological complications in most IEMs are currently unknown. Therefore, there are no pharmacological treatments for neurodevelopmental disorders associated with IEMs. Although causal therapy, dietary restrictions or the supplementation of enzyme cofactors, improves clinical outcomes in some IEM patients, the therapeutic effect on neurodevelopmental disorders is often limited^6,7,12–14^. Moreover, dietary restriction is ineffective for IEMs in which harmful metabolites are generated by endogenous sources, and these treatments are not indicated for the majority of IEMs, for which the exact biochemical basis of the disease is unknown. Pharmacological interventions to restore neural circuits may therefore have broad utility in the treatment of neurological disorders that result from various IEMs. However, an understanding of the altered neural circuitry in each IEM is essential for pharmacological intervention. In this respect, animal models provide valuable opportunities for the investigation of disease mechanisms in IEMs^15,16^.

Aminopeptidase P1 deficiency is an IEM caused by mutations in the *Xpnpep1* gene. As aminopeptidase P1 is a widely distributed metallopeptidase that cleaves the first residue from peptides containing a penultimate proline in various tissues^17,18^, the lack of aminopeptidase P1 activity results in massive urinary excretion of undigested peptides containing a penultimate proline in both humans and mice^19,20^. In addition, neurodevelopmental disorders, such as developmental delay, microcephaly, and epilepsy, have been observed in patients with aminopeptidase P1 deficiency^19,20^. We previously reported that aminopeptidase P1 is predominantly expressed in neurons, compared to glial cells, in the hippocampus, and a disruption of aminopeptidase P1 in mice results in neurodegeneration in the hippocampal CA3 area, hyperactivity, and impaired hippocampus-dependent learning and memory^21,22^. However, the identity and biochemical actions of undigested imino-oligo peptides responsible for neurological complications in Xpnpep1^–/–^ mice are unknown. Despite this, we hypothesized that the characterization and pharmacological restoration of altered neural circuitry would reverse neurological symptoms in the mice. This approach may provide an opportunity to develop more effective treatments for neurological complications in various IEMs as well as valuable insight into the pathological mechanism of IEMs.

In this study, we found that the metabolic dysfunction in aminopeptidase P1 deficiency perturbs NMDAR homeostasis in brain neurons, thereby leading to synaptopathy in the hippocampi of Xpnpep1^–/–^ mice. In addition, chronic treatment with memantine, an NMDAR antagonist approved by the US Food and Drug Administration (FDA) for the treatment of Alzheimer’s disease^23^, improved neurological defects in Xpnpep1^–/–^ mice at the cellular and behavioral levels. These observations indicate that neurological complications in IEMs are treatable by pharmacological intervention, and the restoration of neural circuitry may be an effective treatment for neurological symptoms in patients with IEMs.

## Materials and methods

### Animals

The generation of Xpnpep1 mutant mice and the genotyping of the Xpnpep1 allele have been previously described^20^. Mice were backcrossed with two different inbred strains, C57BL/6J and 129S4/SvJae, for 8–16 generations before use. All experiments were performed on age-matched pairs of Xpnpep1^+/+^ and Xpnpep1^–/–^ mice generated by intercrossing C57BL/6J and 129S4/SvJae heterozygous parents. Animals were housed 4–5 per cage in an animal facility and maintained in a climate-controlled room with free access to food and water under a 12-h/12-h light/dark cycle (lights on at 7:00 AM). Animal maintenance and experiments were conducted in accordance with the guidelines of and approved by the Institutional Animal Care and Use Committee at Seoul National University.

### Histology

Histochemical analyses were performed as described previously^21,22^. Briefly, mice were deeply anesthetized with a mixture of Zoletil (50 mg/kg, intraperitoneally [i.p.]) and xylazine (1 mg/kg, i.p.), transcardially perfused with heparinized (10 U/ml) phosphate-buffered saline (PBS), and fixed with 4% (w/v) paraformaldehyde in PBS. Mouse brains were postfixed in the same fixative for 48 h at 4 °C and cut into 60 μm coronal sections using a vibratome (VT1200S, Leica, Germany). The sections were postfixed in the same fixative for 1 h and permeabilized with 0.3% (v/v) Triton X-100 in PBS for 3 h. The sections were incubated in blocking buffer (5% normal goat serum, 5% horse serum, 5% donkey serum, and 0.5% BSA in PBS) for 2 h, incubated overnight at 4 °C with primary antibodies, and incubated with Cy3- or fluorescein isothiocyanate (FITC)-conjugated secondary antibodies for 2 h. After each step, the sections were rinsed three times for 10 min with PBS. Images were acquired using a confocal laser scanning microscope (LSM510, Zeiss, Germany).

For double immunohistochemical/X-gal staining, formalin-fixed mouse brains were postfixed for 12 h at 4 °C and cut into 100 μm-thick sections using a vibratome. The sections were incubated in X-gal staining solution (5 mM K_3_Fe(CN)_6_, 5 mM K_4_Fe(CN)_6_, 2 mM MgCl_2_, 0.01% deoxycholate, 0.02% NP-40, and 1 mg/mL X-gal in PBS) at 37 °C for 5–8 h and then postfixed for 1 h at 4 °C. The sections were rinsed, permeabilized, blocked, and immunostained with primary and secondary antibodies as described above.

For hematoxylin and eosin (HE) staining, fixed brains were embedded in paraffin using an embedding module (Shandon Histocentre 3, Thermo Scientific, USA) and dissected into 4 μm sections using a rotary microtome (RM2145, Leica), and sections were mounted on glass slides. The sections were deparaffinized and rehydrated by immersing successively in xylene (three times for 10 min), 100% ethanol (twice for 5 min), 95% ethanol (twice for 5 min), and running tap water for 5 min. The sections were then stained with hematoxylin (Merck) and eosin (Sigma–Aldrich). The stained sections were successively rinsed with 95% ethanol, 100% ethanol, and xylene. Images were acquired using a light microscope (BX-51, Olympus) with a digital imaging system (DFC280, Leica).

### Antibodies and western blotting

GluA1 and GluA2 antibodies have been previously described^24^. The following antibodies were purchased commercially: PSD-95 (Thermo Scientific, MA1-045), synapsin I (Chemicon, AB1543), GluN1 (BD Biosciences, 556308), GluN2A (BD Biosciences, 612286), GluN2B (BD Biosciences, 610416), VGLUT1 (Synaptic Systems, 135 303), α-tubulin (Sigma, T5168), NeuN (Millipore, ABN78), MAP2 (Sigma, M9942), p-CaMKII (Abcam, ab32678), and Calpain-1 (Cell Signaling Technology, 2556S).

For western blotting, mouse forebrains or hippocampi were homogenized in homogenization buffer (320 mM sucrose, 10 mM Tris-HCl, 5 mM EDTA, pH 7.4) containing a protease inhibitor cocktail (Sigma–Aldrich, MO, USA, Cat. # P8340) and a phosphatase inhibitor cocktail (GenDEPOT, TX, USA, Cat. # P3200). Homogenates were separated using sodium dodecyl sulfate–polyacrylamide gel electrophoresis, and proteins were transferred to nitrocellulose membranes. After protein transfer, the membranes were incubated in blocking buffer [5% skim milk in Tris-buffered saline with 0.1% Tween 20 (TBST)] for 30 min at room temperature and then successively were incubated with primary antibodies and horseradish peroxidase (HRP)-conjugated secondary antibodies (Jackson ImmunoResearch, West Grove, USA). After each step, the membranes were rinsed three times for 10 min with TBST. The HRP signals were developed using enhanced chemiluminescence (GE Healthcare, UK) and detected by exposing the membrane to X-ray film. Western blot signals were quantified using MetaMorph software (Molecular Devices).

### Slice electrophysiology

**Fig. 1:**
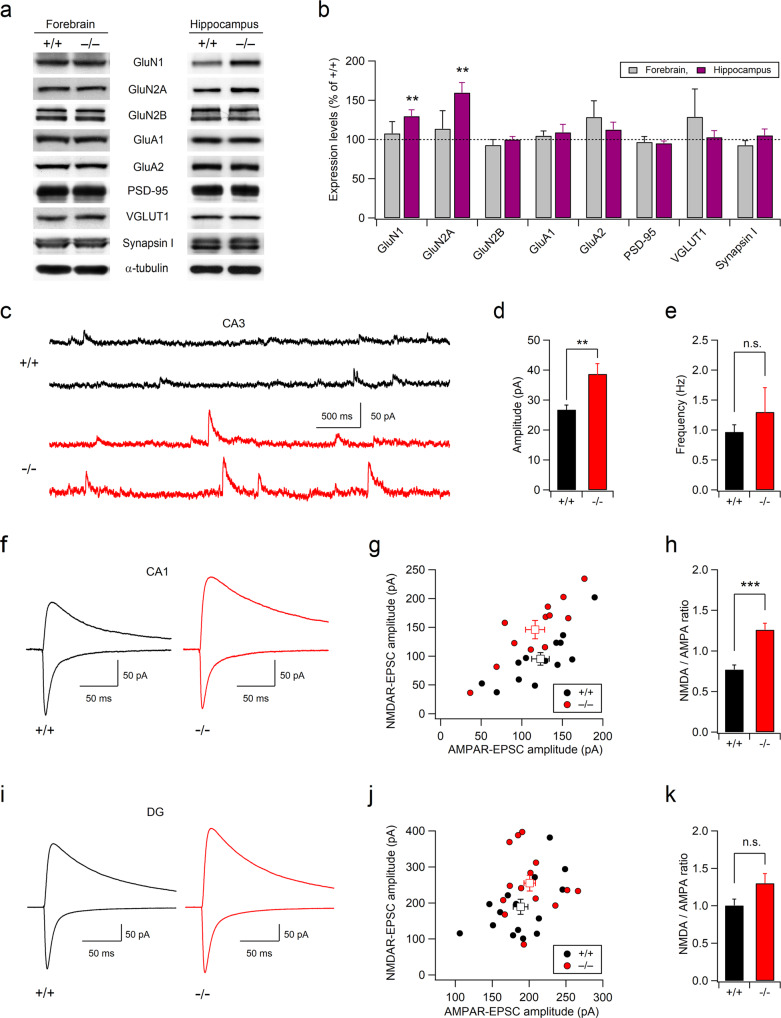
Altered NMDA receptor expression and synaptic transmission in the hippocampi of Xpnpep1^–/–^ mice. **a** Representative western blots for excitatory synaptic proteins in the forebrain and hippocampal homogenates from Xpnpep1^+/+^ and Xpnpep1^–/–^ mice. α-Tubulin was used as a loading control. The blots are cropped, and full blots are shown in the Supplementary Information. **b** Relative expression levels of synaptic proteins in the forebrains (gray bars) and hippocampi (purple bars) of Xpnpep1^–/–^ mice. The signal intensity of each band was normalized to that of α-tubulin, and the expression level of each protein in samples from Xpnpep1^–/–^ mice is expressed as the percentage of the expression level in samples from WT (+/+) mice; *n* = 4 pairs. **c**–**h** Enhanced NMDAR-mediated transmission in CA3 (**c**–**e**) and CA1 (**f**–**h**) pyramidal neurons from Xpnpep1^–/–^ mice. **c** Sample traces of NMDAR-mediated miniature excitatory postsynaptic currents (mEPSCs) recorded at a holding potential of +40 mV in hippocampal CA3 neurons. Mean amplitudes (**d**) and frequencies (**e**) of NMDAR-mEPSCs in CA3 neurons are summarized. Amplitude: *t*_(26)_ = –3.13, *p* = 0.0043; frequency: *t*_(26)_ = –0.78, *p* = 0.44; *n* = 14 cells (3 mice) in each group (**d**, **e**). **f** Sample traces of evoked AMPA receptor (AMPAR)-EPSCs measured at a holding potential of –70 mV (downward deflections) and NMDAR-EPSCs recorded at a holding potential of +40 mV (upward deflections) at SC-CA1 synapses. **g** Mean amplitudes of evoked NMDAR-EPSCs recorded from each neuron are plotted against the mean amplitudes of evoked AMPAR-EPSCs. Squares indicate the mean of values in mice of each genotype. **h** Mean NMDA/AMPA ratios measured at SC-CA1 synapses from WT and Xpnpep1^–/–^ mice. *n* = 13 (+/+) and 12 (–/–) cells from 4 mice. *t*_(23)_ = –4.95 and *p* = 0.000053 by Student’s *t* test. **i**–**k** Normal NMDAR-mediated synaptic transmission in Xpnpep1^–/–^ dentate granule cells. **i** Sample traces of AMPAR- and NMDAR-mediated currents shown as downward (at –70 mV) and upward (at +40 mV) deflections, respectively. **j** Mean amplitudes of NMDAR-EPSCs from individual WT and Xpnpep1^–/–^ granule cells are plotted against the amplitudes of AMPAR-EPSCs. **k** Normal AMPA/NMDA ratios in Xpnpep1^–/–^ dentate granule cells; *n* = 15 cells from 3 mice in each group. *t*_(28)_ = –1.92 and *p* = 0.064 by Student’s *t* test; *U* = 75, *Z* = –1.55, *p* = 0.12 by Mann–Whitney tests.

Electrophysiological recordings from hippocampal slices were performed as previously described^22^. Hippocampal sections (400 μm) from 5-week-old mice of both sexes were prepared using a vibratome (Leica, Germany) in ice-cold dissection buffer (230 mM sucrose; 25 mM NaHCO_3_; 2.5 mM KCl; 1.25 mM NaH_2_PO_4_; 10 mM D-glucose; 1.3 mM Na-ascorbate; 3 mM MgCl_2_; 0.5 mM CaCl_2_, pH 7.4 with 95% O_2_/5% CO_2_). The slices were recovered for at least 1 h at 36 °C in an aerated (95% O_2_, 5% CO_2_) artificial cerebrospinal fluid (ACSF) solution (125 mM NaCl, 26 mM NaHCO_3_, 2.5 mM KCl, 1.25 mM NaH_2_PO_4_, 1.3 mM MgCl_2_, 2.5 mM CaCl_2_, 10 mM D-glucose) and then maintained at room temperature. All electrophysiological experiments were performed in a submerged-type recording chamber, which was perfused with heated (29–30 °C) ACSF. The signals were filtered at 2.8 kHz and digitized at 10 kHz using a MultiClamp 700B amplifier and a Digidata 1440 A interface (Molecular Devices, CA, USA). During the whole-cell patch clamp recording, the series resistance (< 10 MΩ) and seal resistance (> 1 GΩ) were monitored by applying a short (50 ms) hyperpolarization voltage pulse (−5 mV), and the data were discarded if the resistance changed by more than 20%. AMPA receptor (AMPAR)-mediated miniature excitatory postsynaptic currents (mEPSCs) were recorded at –70 mV using a pipette (3–4 MΩ) solution containing 100 mM CsMeSO_4_, 10 mM TEA-Cl, 8 mM NaCl, 10 mM HEPES, 5 mM QX-314-Cl, 2 mM Mg-ATP, 0.3 mM Na-GTP, and 10 mM EGTA (pH adjusted to 7.25 with CsOH, 290 mOsm) in the presence of picrotoxin (50 μM), AP-5 (50 μM), and TTX (0.5 μM) in ACSF. NMDAR-mEPSCs in normal ACSF were recorded at a holding potential of +40 mV (Fig. 1). Picrotoxin, TTX, NBQX (10 μM), and MPEP (10 μM) were added to the bathing solution to inhibit IPSCs, Na^+^ channels, AMPARs, and subtype 5 metabotropic glutamate receptors (mGluR5s), respectively. To determine the effect of memantine on NMDAR activity, NMDAR-mEPSCs were measured at –70 mV, and slices were perfused with Mg^2+^-free ACSF to allow the activation of NMDARs (Fig. 4). For mIPSC recordings at –70 mV, CsMeSO_4_ in the pipette solution was replaced with equimolar CsCl, and NBQX, AP-5, and TTX were added to the normal ACSF.

To measure NMDA/AMPA ratios, synaptic responses were evoked by stimulating Schaffer collaterals with a broken glass pipette (0.3–0.5 MΩ) filled with ACSF. EPSCs were recorded using the same pipette solution used for the measurement of mEPSCs, and stimulation intensity was adjusted to obtain AMPAR-EPSCs with peak amplitudes of 100–250 pA at –70 mV. After recording stable AMPAR-EPSCs for at least 10 min, the AMPAR inhibitor NBQX (10 μM) was added to ACSF, and NMDAR-EPSCs in the same neuron were isolated by +40 mV depolarization. During the AMPAR- and NMDAR-EPSC recordings, picrotoxin (10 μM) was included in the ACSF.

Field excitatory postsynaptic potentials (fEPSPs) were recorded using an ACSF-filled recording pipette placed in the stratum radiatum. Schaffer collaterals were stimulated with an ACSF-filled broken glass pipette (0.3–0.5 MΩ), and the stimulation intensity was adjusted to produce one-third of the maximal synaptic responses. LTP was induced by four episodes of theta burst stimulation (TBS) with interepisode intervals of 10 s. An episode of TBS consisted of ten stimulus trains at 5 Hz, with each stimulus train consisting of four pulses at 100 Hz. Low-frequency stimulation (LFS) consists of 900 stimuli at 1 Hz. Slices displaying an unstable (>10%) baseline (20 min) or changes in the fiber volley were discarded.

All electrophysiology data were analyzed using Clampfit (Molecular Devices, USA) and custom macros written in Igor Pro (WaveMetrics). All chemicals were purchased from Sigma–Aldrich (USA), except for picrotoxin, NBQX, AP-5, MPEP, and DHPG, which were purchased from Tocris (UK).

### Behavior analyses

The open-field test, novel object recognition (NOR) test, and contextual fear conditioning test were performed, as previously described^21^, in male mice between 11:00 AM and 6:00 PM. The object location memory (OLM) test was performed using female mice. Animals were transferred to the behavior testing room for at least 1 h before testing for acclimation. All testing apparatuses were sprayed with 70% ethanol and wiped before the start of each trial.

In the open-field test, each mouse was placed in the center of the open-field apparatus with opaque walls (40 × 40 × 40 cm) and allowed to freely explore for 30 min (Fig. 5) or 1 h (Fig. 8) in a dimly lit room. The behavior of each mouse was video recorded, and the distance traveled in the open field box was calculated using video tracking software (Ethovision XT, Noldus, Netherlands).

The NOR and OLM tests were performed in the same chamber used for the open-field test. During the training and test sessions, the animals were allowed to explore the objects for 10 min, with a 24-h intersession interval. Two identical objects were placed in the chamber during the training session, and one of the objects was replaced with a new object in the test session of the NOR test. The test phase of the OLM test was conducted by moving one of the two familiar objects to a different location in the chamber. The behavior of the animals was recorded, and the duration of exploring objects in each session was manually scored by an experienced experimenter blinded to the mouse genotype and treatment. The preference index (%) in the NOR test was calculated as follows: (time spent exploring the new object)/(total time spent exploring both new and familiar objects) × 100. The preference index in the OLM test was calculated as follows: (time spent exploring the moved object)/(total time spent exploring both moved and unmoved objects) × 100.

For the contextual fear conditioning test, animals were allowed to explore the fear conditioning chamber (Coulbourn Instruments) for 5 min, during which (Pre-CS) the activity of each mouse was monitored. The mice were then exposed to a 2-s foot shock (0.7 mA) and returned to their home cage after 60 s. The next day, the animals were returned to the same fear conditioning chamber, and the activity of each mouse was monitored for 5 min (CS). The freezing time (Fig. 5) was manually scored by an experimenter blinded to the mouse genotype and treatment. The extent of activity suppression (Fig. 8) was measured using video tracking software (Ethovision XT, Noldus, Netherlands) as follows: [(distance moved during pre-CS – distance moved during CS)/(distance moved during pre-CS)].

### Statistical analysis

Statistical analyses were performed using Igor Pro (WaveMetrics) and SPSS (Apache Software Foundation). The collected data were compared using parametric two-tailed Student’s *t* tests or nonparametric Mann–Whitney tests. A one- or two-way analysis of variance (ANOVA) with the Tukey multiple comparison test was used to compare multiple groups. All bar graphs in the figures show the mean ± standard error of the mean (SEM).

## Results

### Deficiency of aminopeptidase P1 causes enhanced NMDAR expression and activity in the hippocampus

**Fig. 2:**
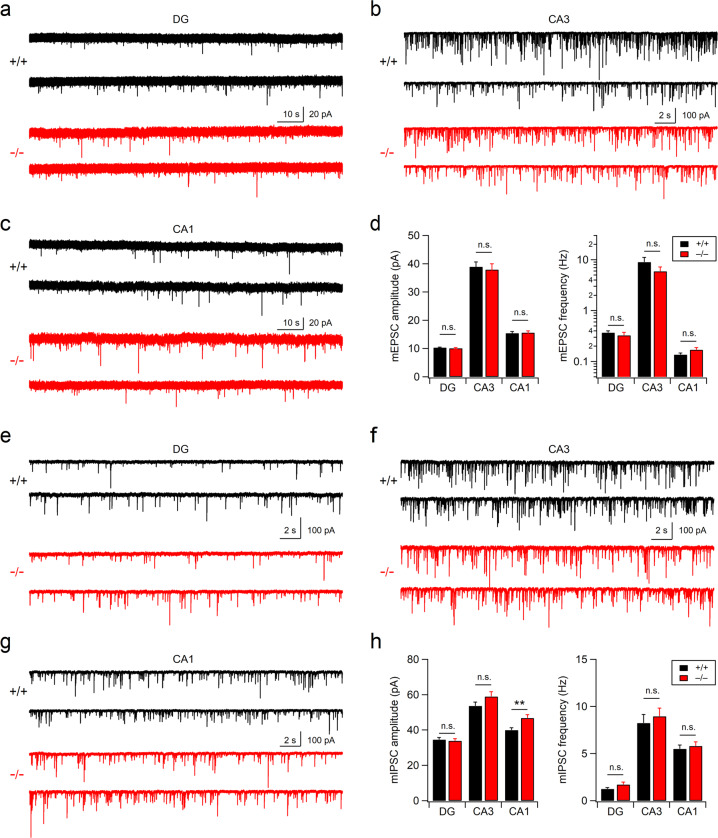
Analysis of AMPAR-mEPSCs and mIPSCs in hippocampal DG, CA3, and CA1 principal neurons from WT and Xpnpep1^–/–^ mice. **a**–**c** Sample traces of AMPAR-mEPSCs measured in dentate granule cells (**a**), CA3 pyramidal neurons (**b**), and CA1 pyramidal neurons (**c**) from WT and Xpnpep1^–/–^ mice. AMPAR-mEPSCs were recorded at a holding potential of –70 mV in the presence of TTX, AP-5, and picrotoxin in the bathing solution. **d** Mean amplitude (left) and frequency (right) of AMPAR-mEPSCs show intact AMPAR-mediated synaptic transmission throughout the hippocampus of Xpnpep1^–/–^ mice. DG; *n* = 18 cells (3 mice) in each group; *t*_(34)_ = 0.83 and *p* = 0.41 for mEPSC amplitude; *t*_(34)_ = 0.74 and *p* = 0.46 for mEPSC frequency. CA3; *n* = 20 cells (3 mice) in each group; *t*_(38)_ = 0.36 and *p* = 0.72 for mEPSC amplitude; *t*_(38)_ = 1.24 and *p* = 0.22 for mEPSC frequency. CA1; *n* = 25 cells (5 mice) in each group; *t*_(48)_ = –0.22 and *p* = 0.82 for mEPSC amplitude; *t*_(48)_ = –1.67 and *p* = 0.10 for mEPSC frequency. Student’s *t* test. **e**–**g** Sample traces of mIPSCs measured in dentate granule cells (**e**), CA3 pyramidal neurons (**f**), and CA1 pyramidal neurons (**g**) from WT and Xpnpep1^–/–^ mice. During the mIPSC recordings, the membrane potential was held at –70 mV in the presence of blockers for NMDARs (AP-5), AMPARs (NBQX), and Na^+^ channels (TTX). **h** Quantification of mIPSC amplitudes (left) and frequencies (right) in DG, CA3, and CA1 principal neurons from WT and Xpnpep1^–/–^ mice. DG; *n* = 18 cells (3 mice) in each group; *t*_(34)_ = 0.39 and *p* = 0.70 for mIPSC amplitude; *t*_(34)_ = –1.50 and *p* = 0.14 for mIPSC frequency; Student’s *t* test. CA3; *n* = 20 cells (3 mice) in each group; *t*_(38)_ = –1.52 and *p* = 0.14 for mIPSC amplitude; *t*_(38)_ = –0.57 and *p* = 0.57 for mIPSC frequency; Student’s *t* test. CA1; *n* = 18 cells (3 mice) in each group; *t*_(34)_ = –2.96 and *p* = 0.0056 by Student’s *t* test, and *U* = 74, *Z* = –2.78, and *p* = 0.005 by Mann–Whitney test for mIPSC amplitude; *t*_(34)_ = –0.51 and *p* = 0.61 by Student’s *t* test, and *U* = 162, *Z* = 0, and *p* = 1.0 by Mann–Whitney test for mIPSC frequency.

To investigate the synaptic mechanisms underlying neurological and behavioral deficits in Xpnpep1^–/–^ mice, we first examined the expression levels of excitatory synaptic proteins in Xpnpep1^–/–^ mouse brains (Fig. 1a, b). We observed a significant increase in the expression levels of the NMDAR subunits GluN1 (*t*_(6)_ = –3.84 and *p* = 0.0086 by Student’s *t* test) and GluN2A (*t*_(6)_ = –3.90 and *p* = 0.0080 by Student’s *t* test) in the Xpnpep1^–/–^ mouse hippocampal homogenates, while forebrain homogenates from Xpnpep1^–/–^ and wild-type (WT, Xpnpep1^+/+^) mice showed similar levels of GluN1 and GluN2A proteins. Interestingly, the expression levels of other excitatory synaptic proteins, including GluN2B, GluA1, GluA2, PSD-95, VGLUT1, and synapsin I, did not change in either homogenate (Fig. 1a, b and Supplementary Fig. 1). Enhanced expression of GluN1 and GluN2A suggests that a deficiency of aminopeptidase P1 increases NMDAR-mediated signaling in the hippocampus. Indeed, a significant enhancement in NMDAR-mediated synaptic transmission was detected in the selected subregions of the hippocampus. NMDAR-mediated miniature excitatory postsynaptic currents (NMDA-mEPSCs) measured in the Xpnpep1^–/–^ CA3 pyramidal neurons at a holding potential of +40 mV in the presence of blockers of AMPARs, GABA_A_ receptors (GABA_A_Rs), voltage-gated Na^+^-channels, and mGluR5s were significantly larger than those measured in WT CA3 neurons, whereas there was no difference in the frequencies of NMDA-mEPSCs between the two genotypes (Fig. 1c–e). A larger amplitude with a normal frequency of NMDA-mEPSCs, in addition to the enhanced hippocampal expression of GluN1 and GluN2A, indicates an increased NMDAR content in each excitatory synapse rather than an increase in the number of silent synapses in Xpnpep1^–/–^ CA3 neurons. Similar to the large NMDA-mEPSCs in CA3 neurons, synaptic NMDA/AMPA ratios, as determined from the evoked NMDAR-EPSCs and AMPAR-EPSCs, at Schaffer collateral (SC)-CA1 synapses were significantly increased in Xpnpep1^–/–^ CA1 pyramidal neurons (Fig. 1f–h). Interestingly, however, the NMDA/AMPA ratios in dentate gyrus (DG) granule cells were not changed by the genetic disruption of aminopeptidase P1 (Fig. 1i–k). To determine whether subregion-specific changes in NMDAR-mediated neurotransmission in the hippocampi of Xpnpep1^–/–^ mice are associated with the expression level of aminopeptidase P1 in each hippocampal subregion, we investigated the expression pattern of aminopeptidase P1 in the hippocampus using X-gal staining, as a β-galactosidase (lacZ) reporter is expressed in Xpnpep1 mutant mice under the control of the Xpnpep1 promoter. Immunohistochemical staining of the neuronal marker NeuN on an X-gal-stained Xpnpep1^+/–^ mouse hippocampal section revealed a distinct pattern of aminopeptidase P1 expression in the principal layer of each hippocampal subregion. In contrast to the CA1 and CA3 areas, in which X-gal signals were detected throughout the neuronal somata in the principal cell layer, the DG exhibited prominent X-gal signals in the middle to outer molecular layers and in the deep layer of the granule cell layer (Supplementary Fig. 2). Intriguingly, most granule cells with somata located in the superficial layer of the granule cell layer did not exhibit X-gal signals in their somata (Supplementary Fig. 2e), indicating that aminopeptidase P1 deficiency is less likely to affect NMDAR activity in these neurons. We further examined whether aminopeptidase P1 deficiency affected AMPAR- or GABA_A_R-mediated synaptic transmission in the hippocampus. AMPAR-mediated synaptic transmission in the CA3, CA1 and DG principal neurons did not change in Xpnpep1^–/–^ mice, as revealed by the normal amplitudes and frequencies of AMPAR-mEPSCs (Fig. 2a–d). This observation suggests that enhanced NMDA/AMPA ratios at SC-CA1 synapses are unlikely to originate from decreased AMPAR currents. In addition, Xpnpep1^–/–^ mice exhibited normal miniature inhibitory postsynaptic currents (mIPSCs) in the CA3 and DG principal neurons, whereas the frequency of mIPSCs, but not the amplitude, in CA1 pyramidal neurons was increased in Xpnpep1^–/–^ mice (Fig. 2e–h). Collectively, these results indicate that a deficiency of aminopeptidase P1 results in synaptic dysfunction, mainly characterized by exaggerated NMDAR signaling, in hippocampal CA3 and CA1 pyramidal neurons.

### Aminopeptidase P1-deficient mice exhibit enhanced NMDAR-dependent long-term potentiation at SC-CA1 synapses

**Fig. 3:**
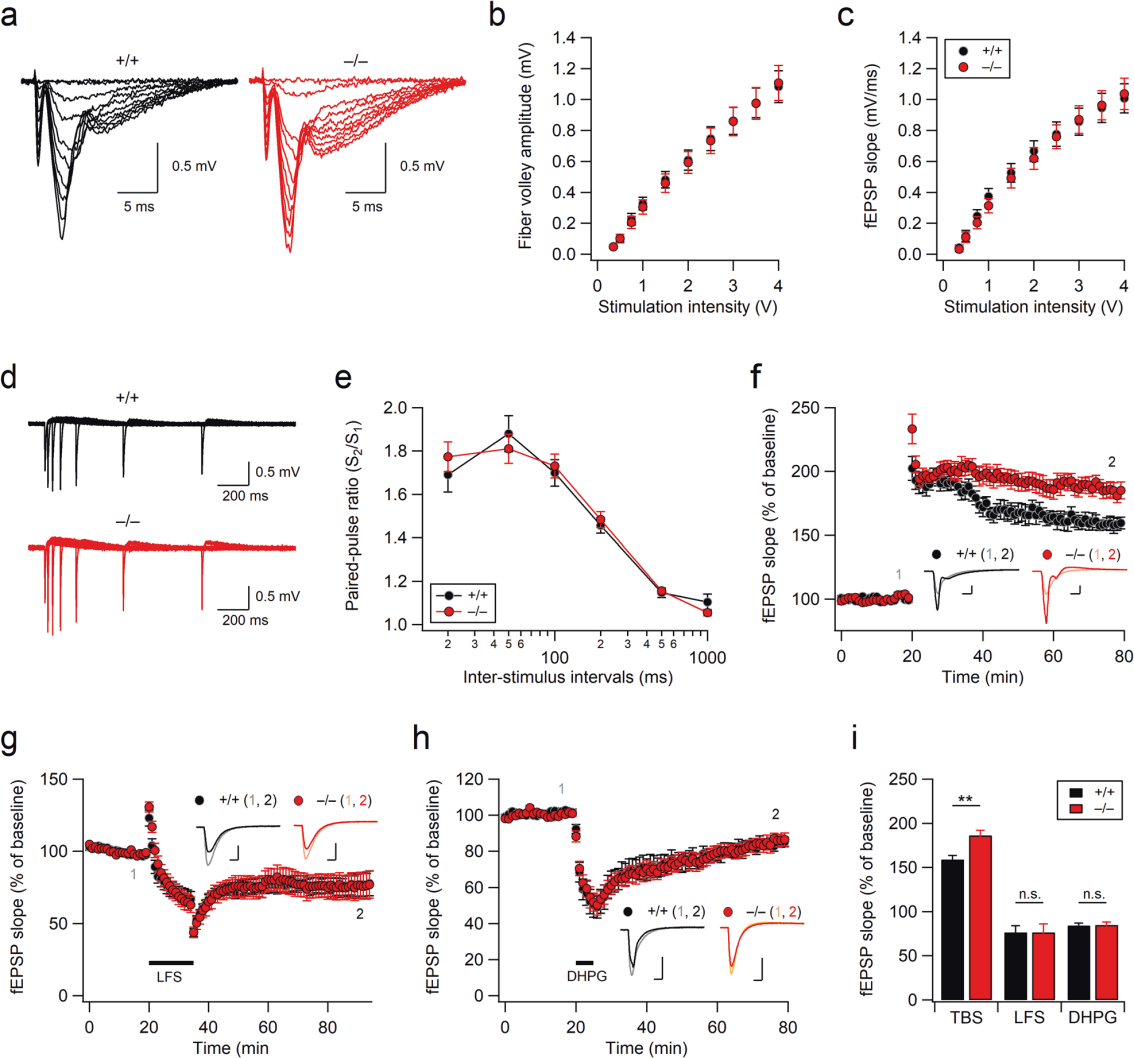
Enhanced NMDAR-dependent LTP at Xpnpep1^–/–^ SC-CA1 synapses. **a**–**c** Xpnpep1^–/–^ mice exhibit normal synaptic input–output relationships across a wide range of stimulus intensities. **a** Sample traces of fEPSPs measured at SC-CA1 synapses in response to incrementally increased stimulus intensities. The amplitudes of the fiber volley (**b**) and the initial slopes of fEPSPs (**c**) are plotted against stimulation intensities. *n* = 13 (+/+) and 15 (−/−) slices from 5 mice. *t*_(26)_ = 0.02, 0.08, 0.38, 0.37, 0.30, 0.21, 0.16, 0.10, 0.11, −0.06, and *p* > 0.05 for fiber volley (FV) amplitudes at 0.35–4 V stimulation intensities; *t*_(26)_ = 0.44, 0.19, 0.76, 0.88, 0.39, 0.47, 0.15, −0.12, −0.12, −0.20, and *p* > 0. 05 for fEPSP slopes at 0.35–4 V stimulation intensities; Student’s *t* tests. **d** Representative traces of fEPSPs evoked by two consecutive stimuli with various interstimulus intervals. **e** Average paired-pulse ratio, the ratio of the second fEPSP slope relative to the first fEPSP slope, is plotted as a function of interstimulus intervals. *n* = 13 (+/+) and 16 (−/−) slices from 5 mice. *t*_(27)_ = −0.80 (20 ms), 0.65 (50 ms), −0.38 (100 ms), −0.52 (200 ms), −0.33 (500 ms), 1.33 (1 s) and *p* > 0.05 by Student’s *t* tests. **f** Theta burst stimulation induced enhanced LTP at Xpnpep1^–/–^ SC-CA1 synapses. The slopes of fEPSPs are normalized to the average baseline response over 20 min before TBS. *n* = 10 slices (5 mice) in each group. **g** Time courses of fEPSP slopes are expressed as a percentage of baseline. Low-frequency stimulation (LFS, 900 stimulations at 1 Hz) induced a similar magnitude of LTD in the hippocampi of WT (10 slices from 5 mice) and Xpnpep1^–/–^ (11 slices from 5 mice) mice. **h** WT and Xpnpep1^–/–^ mice showed similar synaptic responses during and after DHPG (50 μM) perfusion. *n* = 7 slices (4 mice) in each group. (**f**–**h**, insets) Sample traces of fEPSPs obtained at the indicated time points (1 and 2); scale bars, 5 ms and 0.5 mV. Stimulus artifacts and FVs have been removed for clarity (**a**, **d**, and **f**–**h**). **i** Summary bar graphs representing the average normalized fEPSP slopes during the last 10 min of recordings shown in **f**–**h**. *t*_(18)_ = −3.71 and *p* = 0.0015 for TBS; *t*_(19)_ = 0.00056 and *p* = 0.99 for LFS; *t*_(12)_ = −0.14 and *p* = 0.89 for DHPG; **p < 0.01; n.s., not significant (*p* ≥ 0.05) by Student’s *t* tests.

NMDARs play pivotal roles in the induction of activity-dependent modification of synaptic strength, also known as synaptic plasticity, in hippocampal neurons, and alterations in NMDAR activity are often associated with neuropsychiatric disorders. We hypothesized that abnormal NMDAR activity would influence NMDAR-dependent long-term potentiation (LTP) and/or long-term depression (LTD) in Xpnpep1^–/–^ neurons. Therefore, we recorded field excitatory postsynaptic potentials (fEPSPs) at the hippocampal SC-CA1 synapse. The amplitude of the fiber volley (FV) and the slope of the fEPSP evoked by increasing stimulation intensities were not different between WT and Xpnpep1^–/–^ mice (Fig. 3a–c). As AMPARs mediate the fast component of fEPSPs in hippocampal slices, the normal initial slopes of fEPSPs, together with the normal AMPA-mEPSCs, further suggest intact AMPAR function in Xpnpep1^–/–^ CA1 neurons under basal conditions. In addition, paired-pulse ratios determined from the slopes of fEPSPs evoked by two successive stimuli with varying interstimulus intervals did not change at Xpnpep1^–/–^ SC-CA1 synapses (Fig. 3d–e), indicating that basal presynaptic functions remain normal in Xpnpep1^–/–^ mice. However, theta burst stimulation (TBS) at SC-CA1 synapses produced significantly enhanced LTP in Xpnpep1^–/–^ mice (Fig. 3f), although Xpnpep1^–/–^ mice exhibit impaired hippocampus-dependent learning and memory^21^. The enhancement in the magnitude of LTP was observed within 20 min after TBS and was maintained throughout the recording (Fig. 3f).

Next, we examined the effect of aminopeptidase P1 deficiency on the LTD of synaptic transmission at the same synapse. Intriguingly, LTD induced by low-frequency stimulation (LFS), a form of LTD dependent on either the nonionotropic activation or the ionotropic activation of NMDARs, was indistinguishable between Xpnpep1^+/+^ mice and Xpnpep1^–/–^ mice (Fig. 3g). In addition, mice of both genotypes exhibited similar magnitudes of synaptic depression during and after bath application of the group I metabotropic glutamate receptor (mGluR) agonist DHPG (50 μM; Fig. 3h). Together, these results suggest that a deficiency of aminopeptidase P1 results in enhanced LTP at SC-CA1 synapses (Fig. 3i).

### Suppression of NMDARs restores exaggerated LTP and NMDAR activity in Xpnpep1^–/–^ mice

**Fig. 4:**
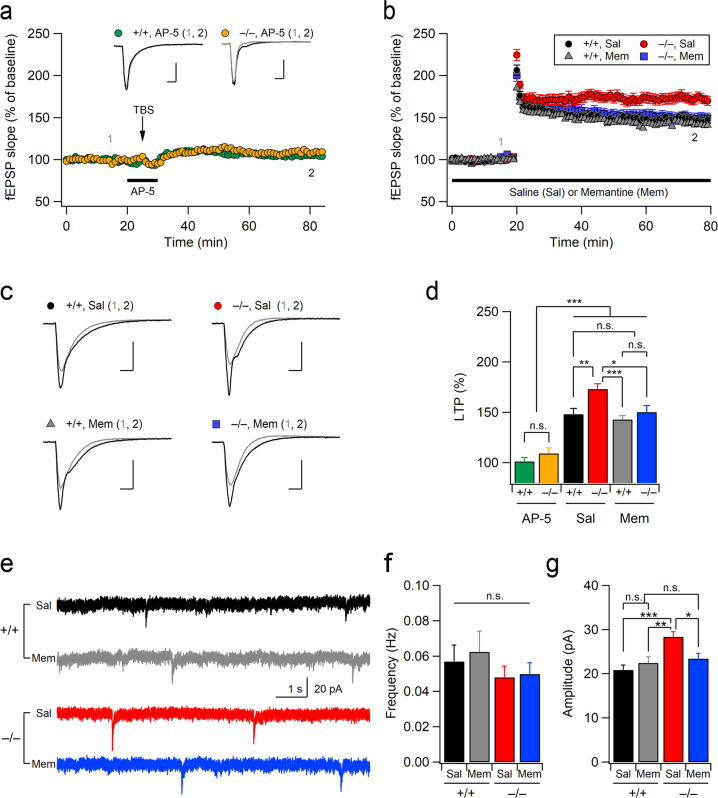
Suppression of NMDAR eliminates the effects of aminopeptidase P1 deficiency on LTP and NMDAR activity. **a** Time courses of fEPSP slopes measured at SC-CA1 synapses are expressed as a percentage of baseline. *n* = 7 slices from 4 mice (+/+, AP-5) and 7 slices from 3 mice (–/–, AP-5). The NMDAR blocker AP-5 (50 μM) was perfused 5 min before and after TBS. (inset) Sample traces of fEPSPs obtained at the indicated time points (1 and 2); Scale bars, 5 ms and 0.5 mV. **b** Normalized fEPSP slope in WT (Xpnpep1^+/+^) and Xpnpep1^–/–^ mice measured in the presence of memantine (Mem, 2 μM) or vehicle (Sal). Memantine significantly reduced the magnitude of LTP in Xpnpep1^–/–^ but not in WT mice. *n* = 15 (+/+, Sal), 14 (–/–, Sal), 13 (+/+, Mem), and 10 (–/–, Mem) slices from 5, 6, 5, and 5 mice, respectively. **c** Sample traces of fEPSP responses during the baseline (1) and final 10 min (2) of recordings shown in b are superimposed. Scale bars, 5 ms and 0.5 mV. Stimulus artifacts and FVs have been removed for clarity. **d** Magnitudes of LTP measured in the last 10 min of recording from all groups are summarized. Genotype, *F*(1, 60) = 7.97, *p* = 0.0064; treatment, *F*(2, 60) = 43.51, *p* < 0.001; interaction, *F*(2, 60) = 1.82, *p* = 0.17. **e** Sample traces of NMDAR-mEPSCs recorded at a holding potential of –70 mV in the hippocampal CA3 pyramidal neurons in Mg^2+^-free bath solution (ACSF). **f**, **g** Mean frequencies (**f**) and amplitudes (**g**) of NMDAR-mEPSCs for each condition are summarized. *n* = 15 (+/+, Sal), 15 (–/–, Sal), 15 (+/+, Mem), and 15 (–/–, Mem) cells from 3, 4, 3, and 4 mice, respectively. **f** Genotype, *F*(1, 56) = 1.526, *p* = 0.221; treatment, *F*(1, 56) = 0.178, *p* = 0.674; interaction, *F*(1, 56) = 0.045, *p* = 0.83. **g** Genotype, *F*(1, 56) = 11.70, *p* = 0.0011; treatment, *F*(1, 56) = 1.82, *p* = 0.183; interaction, *F*(1, 56) = 6.97, *p* = 0.011. **d**, **f**, **g** *p < 0.05; **p < 0.01; ***p < 0.001; n.s., not significant (*p* ≥ 0.05); two-way ANOVA with the Tukey multiple comparison test.

As Xpnpep1^–/–^ mice exhibit enhanced NMDAR activity and TBS-induced LTP, we wondered whether the exaggerated NMDAR activity is associated with abnormal LTP in the Xpnpep1^–/–^ hippocampus. Therefore, we examined the effect of NMDAR inhibition on LTP at SC-CA1 synapses. Bath application of the NMDAR antagonist AP-5 (50 μM) 5 min before and after TBS completely blocked the induction of LTP in mice of both genotypes (Fig. 4a), suggesting that the long-lasting potentiation induced by TBS in our experiments was dependent on NMDAR activation.

If exaggerated NMDAR activity during TBS is responsible for the enhanced LTP and initial potentiation in Xpnpep1^–/–^ mice, the suppression of excessive NMDAR activity would restore abnormal synaptic potentiation. We tested this prediction using the uncompetitive NMDAR antagonist memantine. As memantine has a low affinity for NMDARs and binds only to open channels, this drug, unlike competitive or noncompetitive NMDAR antagonists, preferentially inhibits the pathological overactivation of NMDARs without disturbing their normal activity^25–27^. Indeed, bath application of memantine (2 μM) during electrophysiological recordings normalized the exaggerated LTP, whereas these concentrations of memantine had no effect on TBS-induced LTP in control mice (Fig. 4b–d).

We further examined whether memantine could restore exaggerated NMDAR activity in Xpnpep1^–/–^ CA3 neurons (Fig. 4e–g). Memantine bears a single positive charge under physiological conditions and has a primary binding site overlapping with that of Mg^2+^ in NMDARs, which is thought to be associated with the voltage-dependent effects of memantine on NMDAR inhibition^26,28,29^. Therefore, we examined the effect of memantine (2 μM) on NMDA-mEPSCs at a holding potential of –70 mV with Mg^2+^-free bathing solution. Under these experimental conditions, NMDA-mEPSCs were detected in the CA3 pyramidal neurons of both genotypes with a significantly (+/+, *t*_(27)_ = 7.77, *p* < 0.001; –/–, *t*_(27)_ = 3.19, *p* = 0.0035) reduced frequency (Fig. 4f) compared to depolarization (+40 mV) and normal Mg^2+^ (1.3 mM) in the bathing solution (Fig. 1e). However, genotype differences in the amplitude of NMDA-mEPSCs were still observed. In addition, bath application of memantine (2 μM) during electrophysiological recordings normalized the increased NMDAR-mediated synaptic transmission in Xpnpep1^–/–^ CA3 neurons (Fig. 4g). Collectively, these results indicate that memantine restores exaggerated NMDAR activity and NMDAR-mediated LTP in Xpnpep1^–/–^ mice.

### Acute memantine administration reverses hyperactivity in Xpnpep1^–/–^ mice

**Fig. 5:**
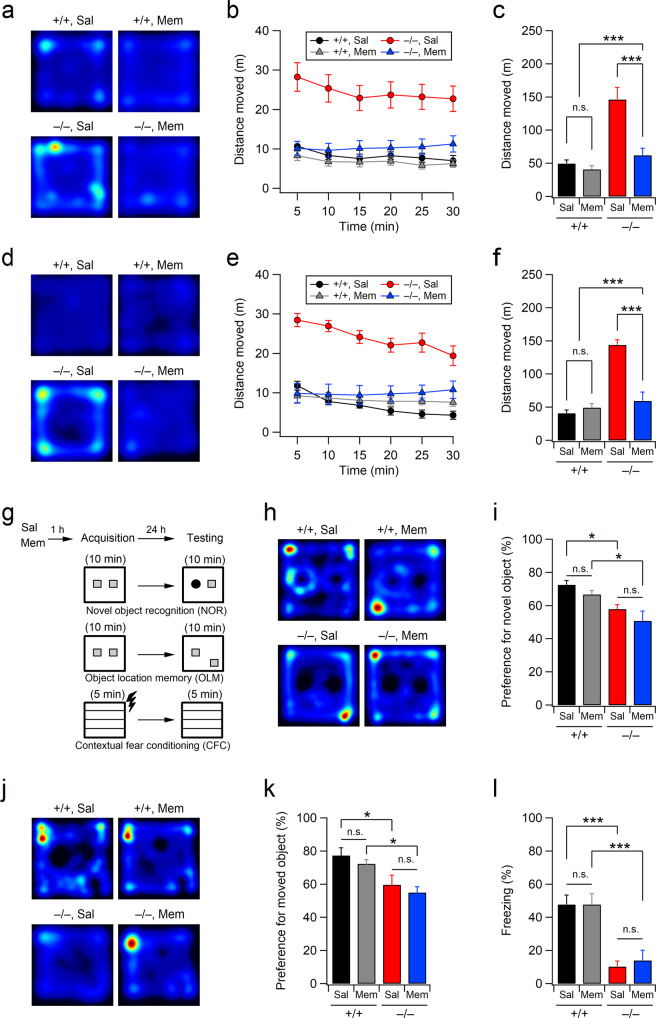
Effects of acute memantine administration on hyperactivity and hippocampus-dependent learning in Xpnpep1^–/–^ mice. **a**–**c** Open-field activities of 6- to 7-week-old male mice were measured 30 min after saline or memantine injection. Representative activity traces during the entire test period (**a**), locomotor activities of mice at 5-min intervals (**b**), and total distance moved during the 30-min open-field test (**c**). *n* = 13 (+/+, Sal), 13 (+/+, Mem), 14 (–/–, Sal), and 12 (–/–, Mem). Genotype, *F*(1, 48) = 24.723, *p* < 0.001; treatment, *F*(1, 48) = 15.375, *p* < 0.001; interaction, *F*(1, 48) = 10.117, *p* < 0.001. **d**–**f** Acute memantine injection reduced hyperactivity in female Xpnpep1^–/–^ mice. Sample path recordings (**d**), distance moved over 5 min time bins (**e**), and locomotor activity during the entire test period (**f**). *n* = 8 (+/+, Sal), 10 (+/+, Mem), 6 (–/–, Sal), and 10 (–/–, Mem). Genotype, *F*(1, 30) = 34.488, *p* < 0.001; treatment, *F*(1, 30) = 15.440, *p* < 0.001; interaction, *F*(1, 30) = 23.255, *p* < 0.001. **g** Experimental designs of the novel object recognition (NOR), object location memory (OLM), and contextual fear conditioning (CFC) tests. Saline or memantine was administered 1 h before the acquisition period. NOR and CFC tests were performed by male mice, and the OLM test was performed by female mice. **h** Representative tracks of movement patterns during the test period of the NOR test. **i** Acute memantine injection had no effect on NOR in male Xpnpep1^–/–^ mice. *n* = 13 (+/+, Sal), 13 (+/+, Mem), 14 (–/–, Sal), and 12 (–/–, Mem). Genotype, *F*(1, 48) = 18.617, *p* < 0.001; treatment, *F*(1, 48) = 3.283, *p* = 0.076; interaction, *F*(1, 48) = 0.032, *p* = 0.86. **j** Representative exploratory tracks of female mice during the test period of the OLM test. **k** Preference index for the moved object in the OLM test. *n* = 8 (+/+, Sal), 7 (+/+, Mem), 7 (–/–, Sal), and 7 (–/–, Mem). Genotype, *F*(1, 25) = 16.357, *p* < 0.001; treatment, *F*(1, 25) = 1.171, *p* = 0.290; interaction, *F*(1, 25) = 0.0008, *p* = 0.98. **l** Percentage of time spent freezing during contextual exposure in the testing period of the CFC test. *n* = 13 (+/+, Sal), 13 (+/+, Mem), 14 (–/–, Sal), and 11 (–/–, Mem). Genotype, *F*(1, 47) = 42.035, *p* < 0.001; treatment, *F*(1, 47) = 0.118, *p* = 0.733; interaction, *F*(1, 47) = 0.128, *p* = 0.722. **c**, **f**, **i**, **k**, and **l**) *p < 0.05; ***p < 0.001; n.s., not significant (*p* ≥ 0.05); two-way ANOVA with the Tukey multiple comparison test.

Xpnpep1^–/–^ mice exhibit hyperactivity and impaired hippocampus-dependent learning^21^. We reasoned that if exaggerated NMDAR signaling is a key mechanism in triggering hyperactivity and cognitive disorders, the suppression of NMDAR activity will reverse the behavioral and cognitive symptoms observed in adult Xpnpep1^–/–^ mice. Indeed, a single intraperitoneal administration of 10 mg/kg memantine induced a significant reduction in both male and female Xpnpep1^–/–^ mouse locomotor activity during the open-field test performed 30 min after drug administration (Fig. 5a–f). This concentration of memantine is known to result in a peak brain concentration of 1–2 μM in rodents^28^. However, a single administration of memantine did not have significant effects on learning and long-term memory in Xpnpep1^–/–^ mice (Fig. 5g–l). Both memantine- and saline-treated Xpnpep1^–/–^ mice showed similar levels of impairment in the NOR and OLM tests, whereas memantine- and saline-treated WT mice preferred novel or moved objects when examined 24 h after administration (Fig. 5h–k). Similarly, acute memantine administration had no effect on the freezing time of Xpnpep1^–/–^ mice during the fear conditioning test (Fig. 5l).

### Chronic memantine treatment reverses neurodegeneration in the hippocampi of Xpnpep1^–/–^ mice

**Fig. 6:**
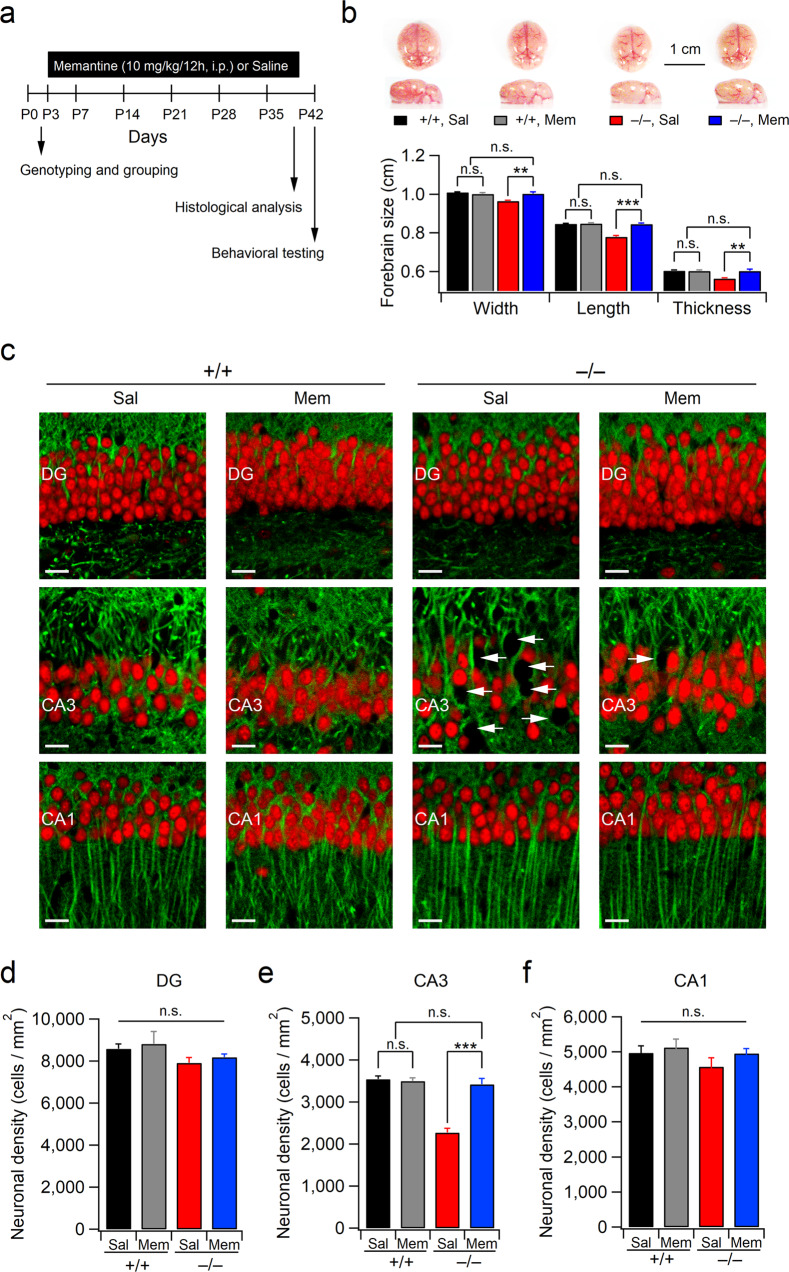
Antagonism of NMDAR protects against neurodegeneration in the hippocampi of Xpnpep1^–/–^ mice. **a** Schematic diagram of the experimental procedure. **b** Memantine rescued microcephaly in Xpnpep1^–/–^ mice. Sample image of brains (top) and the quantification of the brain size (bottom) of 5-week-old mice. *n* = 7 (+/+, Sal), 8 (+/+, Mem), 6 (–/–, Sal), and 6 (–/–, Mem) mice. Width; genotype, F(1, 23) = 11.20, *p* = 0.0027; treatment, *F*(1, 23) = 5.078, *p* = 0.034; interaction, *F*(1, 23) = 13.51, *p* = 0.0012. Length; genotype, *F*(1, 23) = 62.42, *p* < 0.001; treatment, *F*(1, 23) = 55.05, *p* < 0.001; interaction, *F*(1, 23) = 52.61, *p* < 0.001. Thickness; genotype, *F*(1, 23) = 10.32, *p* = 0.0039; treatment, *F*(1, 23) = 8.79, *p* = 0.0069; interaction, *F*(1, 23) = 9.94, *p* = 0.0044. **c** Immunofluorescence images of the hippocampal DG (top), CA3 (middle), and CA1 (bottom) subfields from saline- or memantine-treated WT and Xpnpep1^–/–^ mice. Red, NeuN; Green, MAP2. White arrows indicate the loss of neurons in Xpnpep1^–/–^ mice. Scale bars, 20 μm. **d**–**f** Quantification of neuronal densities in the hippocampal DG (**d**), CA3 (**e**), and CA1 (**f**) subfields. DG; *n* = 8 (+/+, Sal), 6 (+/+, Mem), 8 (–/–, Sal), and 6 (–/–, Mem) slices from three mice in each group; genotype, *F*(1, 24) = 1.14, *p* = 0.296; treatment, *F*(1, 24) = 1.61, *p* = 0.217; interaction, *F*(1, 24) = 1.844, *p* = 0.187. CA3; *n* = 10 (+/+, Sal), 11 (+/+, Mem), 10 (–/–, Sal), and 12 (–/–, Mem) slices from three mice in each group; genotype, *F*(1, 39) = 37.84, *p* < 0.001; treatment, *F*(1, 39) = 25.07, p < 0.001; interaction, *F*(1, 39) = 29.85, *p* < 0.001. CA1; *n* = 7 (+/+, Sal), 6 (+/+, Mem), 6 (–/–, Sal), and 6 (–/–, Mem) slices from three mice in each group; genotype, *F*(1, 21) = 1.19, *p* = 0.285; treatment, *F*(1, 21) = 1.428, *p* = 0.245; interaction, *F*(1, 21) = 4.417, *p* = 0.994. **b**, **d**–**f** **p < 0.01; ***p < 0.001; n.s., not significant (*p* ≥ 0.05); two-way ANOVA with the Tukey multiple comparison test.

Based on the observation that acute memantine administration did not improve learning and long-term memory in Xpnpep1^–/–^ mice, we hypothesized that protection from hippocampal neurodegeneration is required to improve cognitive dysfunction in these mice. We sought to determine whether exaggerated NMDAR activity is associated with CA3 neurodegeneration in Xpnpep1^–/–^ mice^21^. Therefore, we intraperitoneally injected memantine (10 mg/kg) into mice twice daily for five weeks beginning on postnatal day 3 (Fig. 6a). Unexpectedly, chronic memantine treatment improved microcephaly in Xpnpep1^–/–^ mice (Fig. 6b). In contrast to saline-treated Xpnpep1^–/–^ mice, which exhibited prominent reductions in the length and thickness of the forebrain, the brain size of memantine-treated Xpnpep1^–/–^ mice was comparable with that of WT mice that received saline or memantine. In addition, repeated administration of memantine for five weeks prevented early (4–5 weeks old^21^) neuronal cell death in Xpnpep1^–/–^ mice, while saline-treated Xpnpep1^–/–^ mice exhibited severe neurodegeneration with a significant loss of neurons in the hippocampal CA3 but not in the DG and CA1 areas (Fig. 6c-f). These results indicate that perturbations in NMDAR signaling are associated with CA3 neurodegeneration in Xpnpep1^–/–^ mice.

**Fig. 7:**
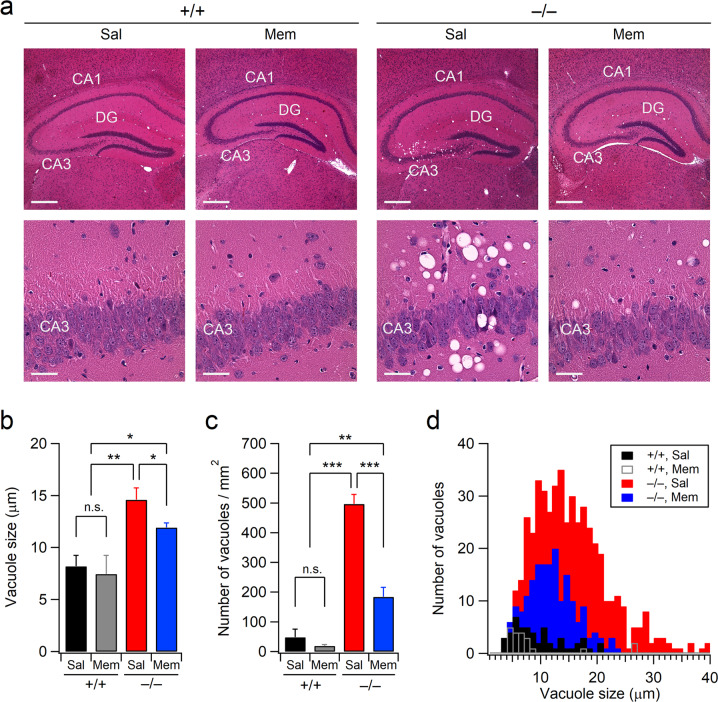
Chronic memantine treatment ameliorates vacuolation in the CA3 subfield of Xpnpep1^–/–^ mice. **a** HE-stained images obtained at lower (top) and higher (bottom) magnifications show a significant reduction in the number of vacuoles in the CA3 region of Xpnpep1^–/–^ mice following memantine treatment. Saline-treated Xpnpep1^–/–^ mice exhibited dark neurons near confluent vacuoles in the CA3 stratum pyramidale. Scale bars, 500 μm (top) and 50 μm (bottom). **b**, **c** Quantification of the size (**b**) and number (**c**) of vacuoles in the hippocampal CA3 subfields. *n* = 4 mice in each group. **b** Genotype, *F*(1, 12) = 19.99, *p* < 0.001; treatment, *F*(1, 12) = 1.96, *p* = 0.18; interaction, *F*(1, 12) = 0.646, *p* = 0.43. **c** Genotype, *F*(1, 12) = 132.17, *p* < 0.001; treatment, *F*(1, 12) = 41.07, *p* < 0.001; interaction, *F*(1, 12) = 28.158, *p* < 0.001; two-way ANOVA with the Tukey multiple comparison test. **d** Histogram shows the size distribution of vacuoles in the hippocampal CA3 subfields. Data were pooled from four mice (12 slices) in each group.

As neurodegeneration in the CA3 region of Xpnpep1^–/–^ mice is accompanied by confluent vacuoles of varying sizes (5–40 μm) with dark surrounding neurons^21^, we further tested whether the restoration of NMDAR activity would suppress vacuolation in the CA3 regions of Xpnpep1^–/–^ mice. Consistent with our previous histochemical observations in the hippocampi of naïve Xpnpep1^–/–^ mice, numerous confluent vacuoles were detected in the hippocampi of saline-treated Xpnpep1^–/–^ mice (Fig. 7a). However, vacuolization was significantly attenuated by repeated memantine administration, as revealed by the reduction in vacuole numbers and size in hematoxylin and eosin (HE)-stained brain sections from memantine-treated Xpnpep1^–/–^ mice (Fig. 7b–d). These results demonstrate that the suppression of exaggerated NMDAR signaling reverses paraptosis-like cell death in CA3 neurons caused by aminopeptidase P1 deficiency.

### Chronic memantine treatment improves the neurodevelopmental and cognitive deficits of Xpnpep1^–/–^ mice

**Fig. 8:**
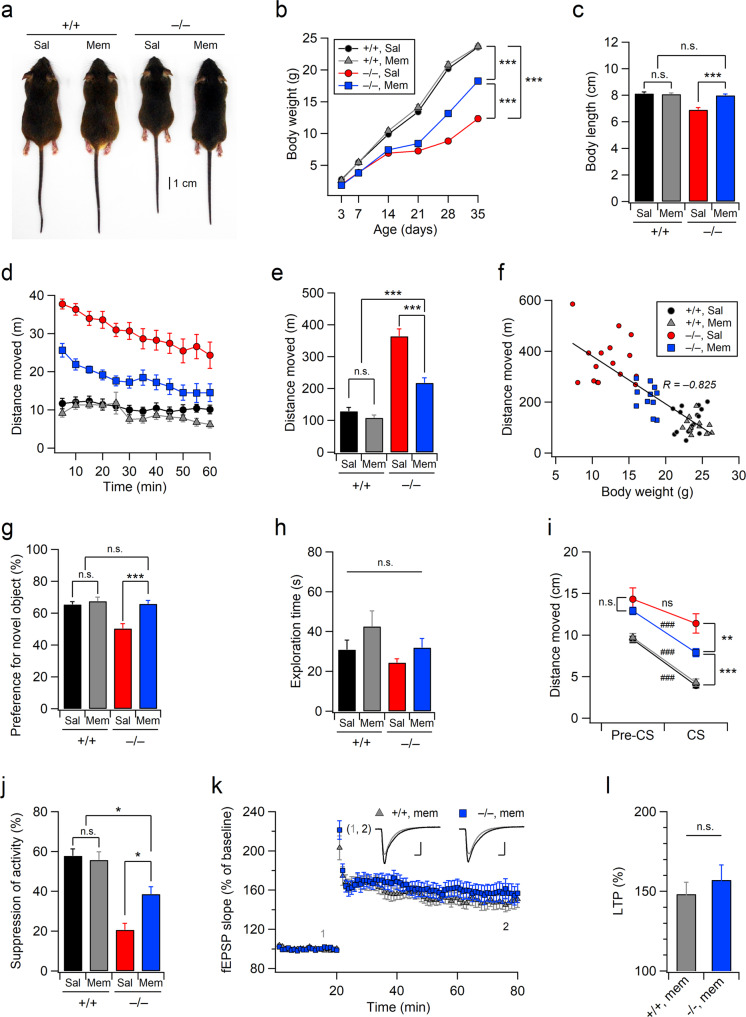
Chronic memantine treatment improves the neurodevelopmental and behavioral deficits of Xpnpep1^–/–^ mice. **a** Rescue of developmental delay by memantine. Representative image of male mice at 5 weeks old. **b** Weight curves of male mice. *n* = 33 (+/+, sal), 20 (+/+, mem), 26 (–/–, sal), and 14 (–/–, mem) mice. Genotype, F(1, 89) = 471.29, *p* = 0; treatment, *F*(1, 89) = 61.24, *p* < 0.001; interaction, *F*(1, 89) = 56.83, *p* < 0.001; at 5 weeks old. **c** Averaged body lengths of male mice at 5 weeks old in each group are summarized. *n* = 7 (+/+, Sal), 8 (+/+, Mem), 6 (–/–, Sal), and 6 (–/–, Mem) mice. Genotype, *F*(1, 23) = 28.10, *p* < 0.001; treatment, *F*(1, 23) = 17.52, *p* < 0.001; interaction, *F*(1, 23) = 32.26, *p* < 0.001. **d** Open-field activities of mice in each group with 5-min intervals. *n* = 16 (+/+, sal; black circles), 12 (+/+, mem; gray triangles), 16 (–/–, sal; red circles), and 11 (–/–, mem; blue squares) mice. **e** Quantification of activity during the entire 1-h period in the open field test. Genotype, *F*(1, 51) = 99.346, *p* < 0.001; treatment, *F*(1, 51) = 23.427, *p* < 0.001; interaction, *F*(1, 51) = 13.175, *p* < 0.001. **f** Plots of open-field activity against body weight show a strong correlation between hyperactivity and developmental delay. “R” represents the Pearson’s correlation coefficient. **g**–**j** Chronic memantine treatment improved hippocampus-dependent learning and memory in Xpnpep1^–/–^ mice. The preference for a new object (**g**) and total exploration time (**h**) in the NOR test. *n* = 11 (+/+, sal), 7 (+/+, mem), 8 (–/–, sal), and 10 (–/–, mem) mice. **g** Genotype, *F*(1, 32) = 12.20, *p* = 0.0014; treatment, *F*(1, 32) = 13.25, *p* < 0.001; interaction, *F*(1, 32) = 7.82, *p* = 0.0086. **h** Genotype, *F*(1, 32) = 2.81, *p* = 0.103; treatment, *F*(1, 32) = 3.496, *p* = 0.070; interaction, *F*(1, 32) = 0.167, *p* = 0.685. **i** Quantification of locomotor activity during contextual fear conditioning tests. *n* = 13 (+/+, sal), 10 (+/+, mem), 8 (–/–, sal), and 10 (–/–, mem) mice. ###*p* < 0.001; ns, not significant (*p* ≥ 0.05) by Mann–Whitney tests. *U* = 0 and *Z* = –4.33 (+/+, sal); *U* = 0 and *Z* = –3.78 (+/+, mem); *U* = 20 and *Z* = –1.26 (–/–, sal); *U* = 1 and *Z* = –3.70 (–/–, mem). Distance moved during CS; genotype, *F*(1, 37) = 85.274, *p* < 0.001; treatment, *F*(1, 37) = 6.867, *p* = 0.012; interaction, *F*(1, 37) = 10.33, *p* = 0.0027. **j** Extent of activity suppression in each group is summarized. Genotype, *F*(1, 37) = 51.81, *p* < 0.001; treatment, *F*(1, 37) = 4.476, *p* = 0.041; interaction, *F*(1, 37) = 7.019, *p* = 0.011. **b**, **c**, **e**, **g**–**j** *p < 0.05; **p < 0.01; ***p < 0.001; n.s., not significant (*p* ≥ 0.05); two-way ANOVA with the Tukey multiple comparison test. **k** Slopes of fEPSPs measured at the hippocampal SC-CA1 synapse from WT and Xpnpep1^–/–^ mice that underwent chronic memantine treatment are plotted against time as a percentage of baseline. *n* = 9 slices from three mice of each genotype. (inset) Sample traces of fEPSPs obtained at the indicated time points (1 and 2); Scale bars, 5 ms and 0.5 mV. **l** Magnitudes of LTP measured during the last 5 min of recording are summarized. *t*_(16)_ = −0.754 and *p* = 0.461 by Student’s *t* test.

As chronic memantine treatment reversed neurodegeneration in the hippocampi of Xpnpep1^–/–^ mice, we investigated whether the behavioral and cognitive deficits in Xpnpep1^–/–^ mice were reversed by chronic memantine treatment. Intriguingly, repeated administration (twice daily) of memantine (10 mg/kg, i.p.) for five weeks ameliorated the developmental delay observed in Xpnpep1^–/–^ mice (Fig. 8a–c). Although memantine-treated Xpnpep1^–/–^ mice gained less body weight than WT mice, a treatment-specific difference in body weight was observed in Xpnpep1^–/–^ mice (Fig. 8b). Memantine had a more profound effect on the body growth of Xpnpep1^–/–^ mice, such that memantine-treated Xpnpep1^–/–^ mice had body lengths similar to those of saline- or memantine-treated Xpnpep1^+/+^ mice at 5 weeks. In addition, long-term treatment with memantine improved hyperactivity in 6-week-old Xpnpep1^–/–^ mice, as shown by the significantly reduced distance traveled in the open-field tests (Fig. 8d, e). Notably, the distance traveled in the open-field arena was inversely correlated (*R* = –0.825) with body weight (Fig. 8f), indicating that hyperactivity during and after weaning might have influenced feeding and energy consumption in Xpnpep1^–/–^ mice.

We next examined the effects of chronic memantine treatment on cognitive dysfunction. In the NOR test, memantine-treated Xpnpep1^–/–^ mice, but not saline-treated Xpnpep1^–/–^ mice, showed levels of new object preference that were comparable to those of WT mice (Fig. 8g, h). Moreover, chronic memantine treatment significantly improved deficits in contextual fear learning in Xpnpep1^–/–^ mice (Fig. 8i, j). As the improved performance of memantine-treated Xpnpep1^–/–^ mice in the fear conditioning test may stem from reduced hyperactivity and enhanced learning, we evaluated the effects of chronic memantine treatment on the performance of Xpnpep1^–/–^ mice in the fear conditioning test using the suppression of activity, an index of adaptive defense reaction^30,31^. There was a significant difference in the suppression of activity between saline- and memantine-treated Xpnpep1^–/–^ mice, indicating that fear learning was improved following chronic memantine treatment (Fig. 8i, j).

We further investigated whether chronic memantine treatment restored NMDAR-dependent LTP and NMDAR expression in the hippocampi of Xpnpep1^–/–^ mice. Intriguingly, chronic memantine treatment abolished genotype-dependent differences in NMDAR-dependent LTP at SC-CA1 synapses (Fig. 8k, l), while this treatment had no effect on the expression levels of hippocampal GluN1 or GluN2A in Xpnpep1^–/–^ mice (Supplementary Fig. 3). However, the downstream signaling activity of NMDARs associated with NMDAR-dependent LTP and neurodegeneration was significantly reduced in Xpnpep1^–/–^ mice that received chronic memantine treatment (Supplementary Fig. 4). Collectively, these results suggest that chronic memantine treatment improves learning and memory in Xpnpep1^–/–^ mice through the suppression of exaggerated NMDAR activity and the restoration of signaling downstream of NMDAR activation.

## Discussion

The present study demonstrates the pathological mechanisms of and a therapeutic strategy for neurological and cognitive disorders in a mouse model of an inborn error of metabolism (IEM) caused by aminopeptidase P1 deficiency. Excessive NMDAR activity in aminopeptidase P1 deficiency induces exaggerated LTP in the hippocampus and triggers neurodevelopmental disorders involving neurodegeneration, hyperactivity, and cognitive dysfunction. These observations indicate that the metabolic dysfunction in aminopeptidase P1 deficiency perturbs NMDAR homeostasis in neurons, thereby leading to synaptopathy. Although aminopeptidase P1 deficiency is a rare IEM in humans^19^, our study provides experimental evidence showing that neurodevelopmental and cognitive deficits in IEMs are treatable and preventable by pharmacological intervention restoring neural circuits.

It has been reported that abnormal NMDAR signaling is also implicated in other IEMs, such as phenylketonuria and homocystinuria^32,33^. Notably, mild to severe intellectual disability and hyperactivity are frequently observed in patients with these IEMs^34–36^. Although the effects of L-phenylalanine at the concentration (0.1–0.4 mM) observed in the brains of patients with phenylketonuria on NMDAR activity are unknown, the plasma concentration (> 1 mM) of L-phenylalanine in patients with phenylketonuria inhibits NMDARs, and chronic exposure to high concentrations of L-phenylalanine upregulates the density of NMDARs in Pah^enu2^ brains with enhanced expression of GluN1 and GluN2A subunits and decreased GluN2B expression^33,37,38^. Similarly, homocysteine modulates both the amplitudes and the desensitization of NMDAR currents dependent on GluN2 subunit composition^39^. Consistent with this, the NMDAR antagonists memantine and MK-801 block the homocysteine-induced cell death of cultured neurons and glia^32^. Whether the restoration of NMDAR signaling, alone or in combination with dietary management, improves intellectual disability and hyperactivity in patients with these diseases warrants further investigation.

Based on the enzymatic action and hippocampal expression pattern of aminopeptidase P1, the metabolic substrates that cause hippocampal dysfunction in Xpnpep1^–/–^ mice are presumably oligopeptides with a penultimate proline^17,19,40^, and they are likely to be cleared in neuronal somatodendritic compartments under normal conditions^22^. It has been reported that the tripeptide glycine-proline-glutamate (GPE) and the tetrapeptide threonine-proline-proline-threonine (TPPT or GLYX-13) act as an NMDAR agonist and modulator, respectively^41,42^. Endogenous cleavage of insulin-like growth factor I (IGF-I) in the brain produces truncated IGF-1 and the N-terminal tripeptide GPE, which binds to the glutamate binding site in NMDARs^43^. High concentrations of GPE activate NMDARs, and the GPE-induced currents are blocked by the competitive NMDAR antagonist 3-((R)-2-carboxypiperazin-4-yl)-propyl-1-phosphonic acid [(R)-CPP] or by extracellular Mg^2+^ at −70 mV^42^. Meanwhile, the tetrapeptide TPPT binds to the glycine modulatory site in NMDARs, thereby acting as a partial agonist of NMDARs, similar to glycine, D-serine, and D-cycloserine^41^. Interestingly, GPE and TPPT possess a proline residue in the second position from the N-terminus and are potential substrates of aminopeptidase P1. The proteolytic degradation of proteins, including proline-rich protein collagen, which consists of glycine-proline-hydroxyproline tripeptide repeats^44^, may produce diverse oligopeptides with a penultimate proline by the action of many proteases or peptidases^40^. It is likely that the accumulation of these peptides in the aminopeptidase P1 deficiency may stimulate or modulate NMDARs in the brain. However, the identity of harmful substrates that perturb NMDAR homeostasis in Xpnpep1^–/–^ mice remains unknown.

Xpnpep1^–/–^ mice exhibited enhanced NMDAR-dependent LTP at SC-CA1 synapses, possibly because of the exaggerated NMDAR activity in CA1 neurons (Figs. 3 and 4), but these mice displayed a significant impairment in hippocampus-dependent learning and memory^21^. The enhanced LTP in Xpnpep1^–/–^ mice appears to be contradictory to their behavioral outcomes (Figs. 5 and 8), as LTP is considered to be one of multiple neuronal learning and memory mechanisms^45,46^. Similar to our study, multiple studies observed an inverse correlation between LTP and learning and memory in genetically engineered mice. Genetic disruption of AMPA receptor 2 (GluA2), postsynaptic density-95 protein (PSD-95), protein tyrosine phosphatase δ (PTPδ), fragile XE-associated familial mental retardation protein 2 (FMR2), and insulin receptor tyrosine kinase substrate p53 (IRSp53) results in enhanced LTP but impaired hippocampus-dependent learning and memory in mice^24,47–50^. As excessive LTP may cause subnormal place cell function^51^, the saturation of LTP^52^, or alterations in the spatial pattern of synaptic weights^53^, abnormally enhanced LTP in the hippocampi of Xpnpep1^–/–^ mice might affect new memory encoding or the accuracy of memory retrieval^45^. Although a low concentration of memantine reversed abnormally enhanced NMDAR activity and NMDAR-dependent LTP in hippocampal slices from Xpnpep1^–/–^ mice (Fig. 4), a single administration of memantine did not improve learning and long-term memory but reversed hyperactivity in Xpnpep1^–/–^ mice (Fig. 5). These observations imply that the impaired hippocampus-dependent learning in Xpnpep1^–/–^ mice is attributable to multiple defects, including severe neurodegeneration in the CA3 area. In support of this idea, chronic memantine administration ameliorated neurodegeneration (Fig. 6) and the impairment of learning and memory (Fig. 8) in Xpnpep1^–/–^ mice. The reduced activation of signaling downstream of NMDARs (Supplementary Fig. 4) and attenuated vacuolation (Fig. 7) in the hippocampi of Xpnpep1^–/–^ mice through chronic memantine treatment further indicate that excessive NMDAR activity during the developmental period is associated with neurodegeneration in CA3 neurons. As CA3 pyramidal neurons receive robust excitatory synaptic inputs, compared to DG or CA1 neurons (Fig. 2), through the mossy fiber-CA3, associational/commissural-CA3, and entorhinal cortex-CA3 pathways^54,55^, excessive NMDAR activity might result in excitotoxic cell death and a disruption of normal ensemble patterns of CA3 neurons by abnormal amplification of synchronous synaptic inputs in dendrites^56,57^. Therefore, excessive NMDAR activity as well as CA3 neurodegeneration and enhanced LTP seem to be associated with impaired learning and memory in Xpnpep1^–/–^ mice.

Interestingly, while the neuronal cell death in the CA3 area of Xpnpep1^–/–^ mice was almost completely prevented by chronic memantine treatment, vacuolation and behavioral outcomes did not fully recover. One possible explanation for this incomplete recovery may be the fast clearance of memantine in mice. The half-life of memantine in mice (<4 h) is much shorter than that in humans (60–80 h), and mice exhibit a steep Cmax/Cmin ratio of up to 100 for memantine^58^. Accordingly, memantine treatment twice daily might be insufficient for the complete correction of behavioral abnormalities in mice. Nevertheless, chronic memantine treatment significantly improved behavioral hyperactivity and impaired learning in Xpnpep1^–/–^ mice.

Although the detailed mechanism by which a deficiency of aminopeptidase P1 perturbs NMDAR homeostasis remains to be elucidated in future studies, the results of the present study suggest that the restoration of neural circuits by pharmacological intervention may be an effective strategy for the treatment of neurodevelopmental, behavioral, and cognitive deficits in patients with IEMs.

## Supplementary information

Supplementary Information
