# Supplementary material for: Suppression of exaggerated NMDAR activity by memantine treatment ameliorates neurological and behavioral deficits in aminopeptidase P1-deficient mice
Source: Exp Mol Med. 2022 Aug 3;54(8):1109–24. doi: 10.1038/s12276-022-00818-9 (PMC9440093; doi:10.1038/s12276-022-00818-9)
Supplement: Supplementary file 1 — Supplementary Information [file 12276_2022_818_MOESM1_ESM.pdf]

Supplementary Information

Suppression of exaggerated NMDAR activity by memantine treatment ameliorates neurological and behavioral deficits in aminopeptidase P1-deficient mice

Young-Soo Bae[1,*], Sang Ho Yoon[1,2,*], Young Sook Kim[1,*], Sung Pyo Oh[1], Woo Seok Song[1,2], Jin Hee Cha[1], and Myoung-Hwan Kim[1,2,3,#]

[1]Department of Physiology and Biomedical Sciences, Seoul National University College of Medicine, Seoul, 03080, Korea. [2]Neuroscience Research Institute, Seoul National University Medical Research Center, Seoul, 03080, Korea. [3]Seoul National University Bundang Hospital, Seongnam, Gyeonggi, 13620, Korea.

*These authors contributed equally to this work.

# To whom correspondence should be addressed; E-mail: kmhwany@snu.ac.kr

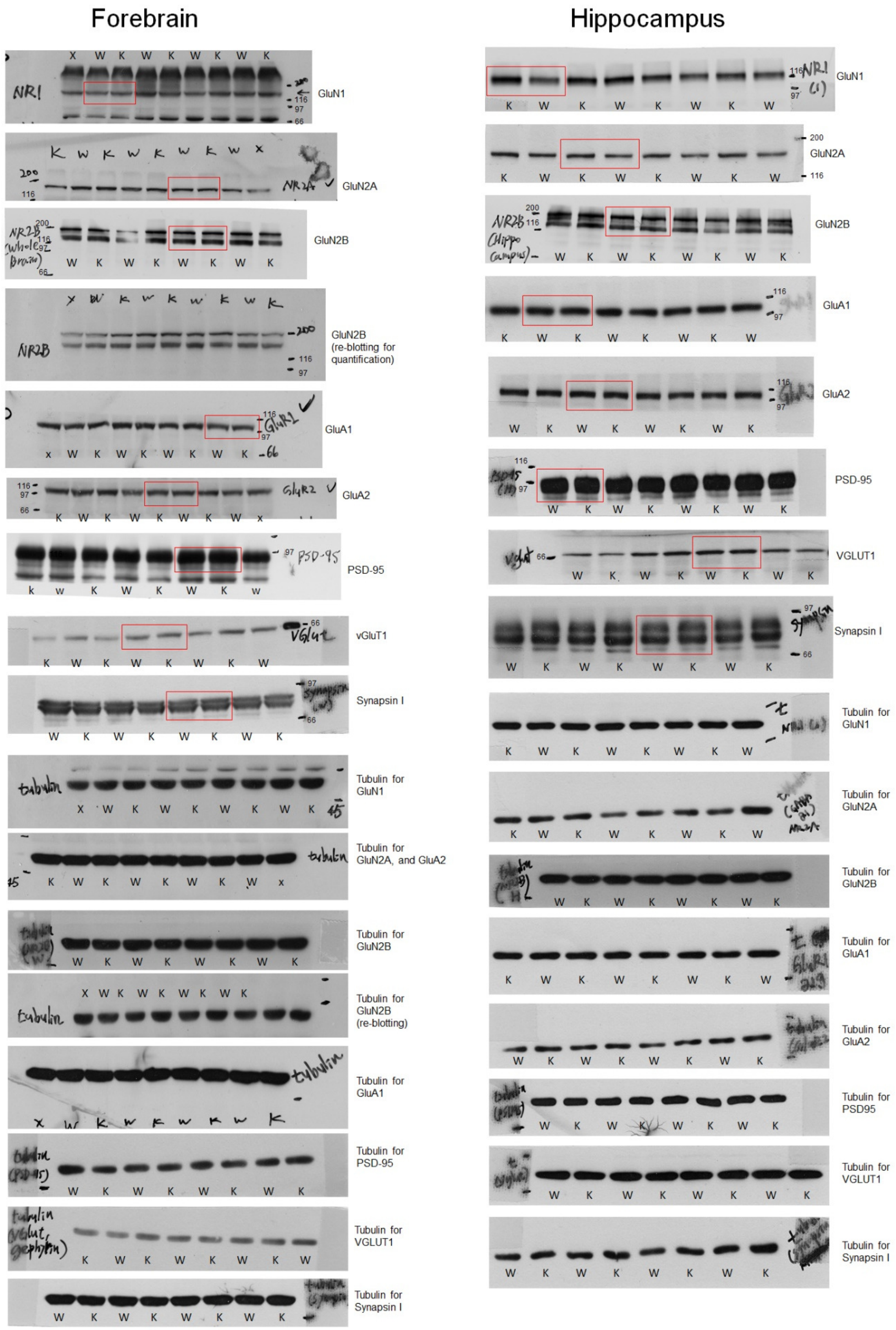

Supplementary Fig. 1. Uncropped western blot images presented in Fig. 1a.

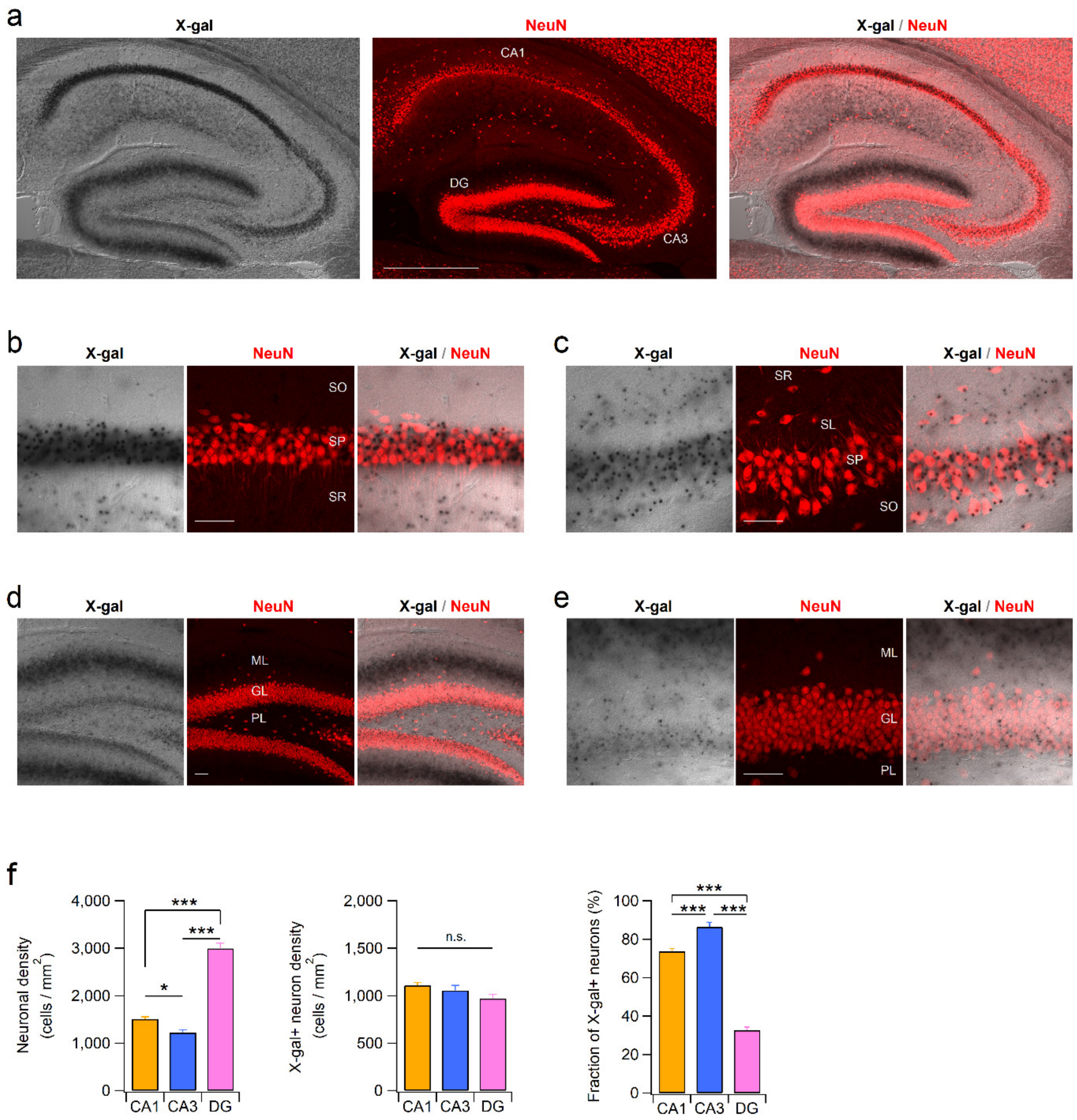

Supplementary Fig. 2. The distribution of X-gal precipitates indicates the expression pattern of aminopeptidase P1 in the Xpnpep1$^{+/-}$ hippocampus. (a) The X-gal-stained sections from Xpnpep1$^{+/-}$ mice were immunostained with NeuN to visualize neuronal somata in the hippocampus. Scale bar, 500 µm. (b, c) Higher magnification views of CA1 (b) and CA3 (c) areas show strong X-gal signals in the somata of neurons in the principal cell layer. (d) DG exhibits strong X-gal signals in the outer molecular layer and moderate X-gal signals in the deep layer of the granule cell layer. (e) Higher magnification view of the granule cell layer. X-gal precipitates in the granule cell layer were mainly detected in the somata of granule cells in the deep layer of the granule cell layer. (b-e) Scale bars, 50 µm. SO, stratum oriens; SP, stratum

pyramidale; SR, stratum radiatum; SL, stratum lucidum; ML, molecular layer; GL, granule cell layer; PL, polymorphic layer. (f) Quantification of NeuN-positive cell density (left), X-gal-positive neuron (X-gal+ and NeuN+) density (middle), and the fraction of X-gal-positive neurons (right) in the principal cell layer of each hippocampal subregion. n = 12 slices from 6 mice. Neuronal density, $F(2, 33) = 154.779$, $p < 0.001$; X-gal+ neuron density, $F(2, 33) = 2.5$, $p = 0.097$; fraction, $F(2, 33) = 217.766$, $p < 0.001$ by one-way ANOVA with the Tukey multiple comparison test.

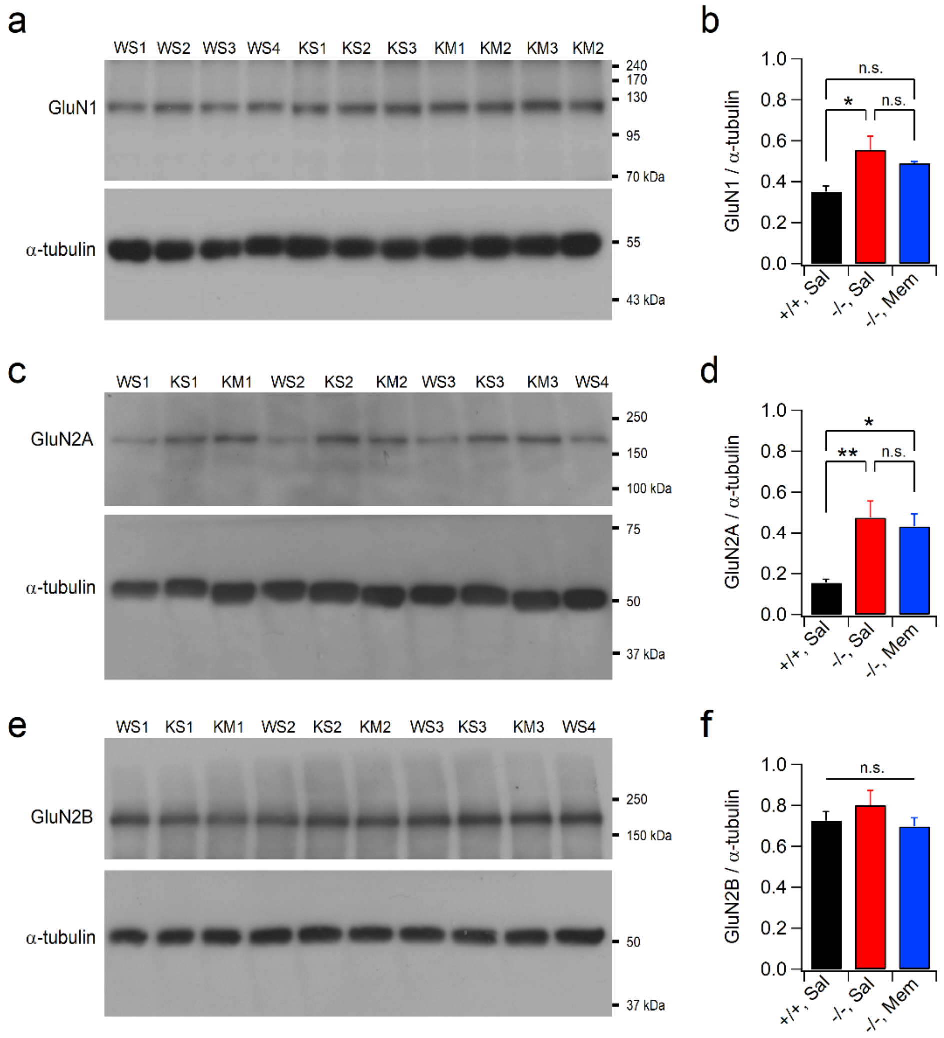

Supplementary Fig. 3. The expression levels of GluN1, Glu2A, and GluN2B in the Xpnpep1$^{-/-}$ hippocampus were not changed by chronic memantine treatment. (a, c, e) Western blots of GluN1 (a), GluN2A (c), and GluN2B (e) in the hippocampal homogenate from Xpnpep1$^{+/+}$ and Xpnpep1$^{-/-}$ mice treated with saline or chronic memantine. WS: saline-treated Xpnpep1$^{+/+}$; KS: saline-treated Xpnpep1$^{-/-}$; KM: memantine-treated Xpnpep1$^{-/-}$. (b, d, f) Quantification of GluN1 (b), GluN2A (d), and GluN2B (f) expression levels in the hippocampus. The signal intensity of each band was normalized to those of α-tubulin. GluN1, $F(2, 7) = 7.272$, $p = 0.019$; GluN2A, $F(2, 7) = 11.568$, $p = 0.006$; GluN2B, $F(2, 7) = 0.933$, $p = 0.437$. *$p < 0.05$; **$p < 0.01$; n.s., not significant ($p \geq 0.05$); one-way ANOVA with the Tukey multiple comparison test. n = 4 (+/+, Sal), 3 (−/−, Sal), and 3 (−/−, Mem) mice.

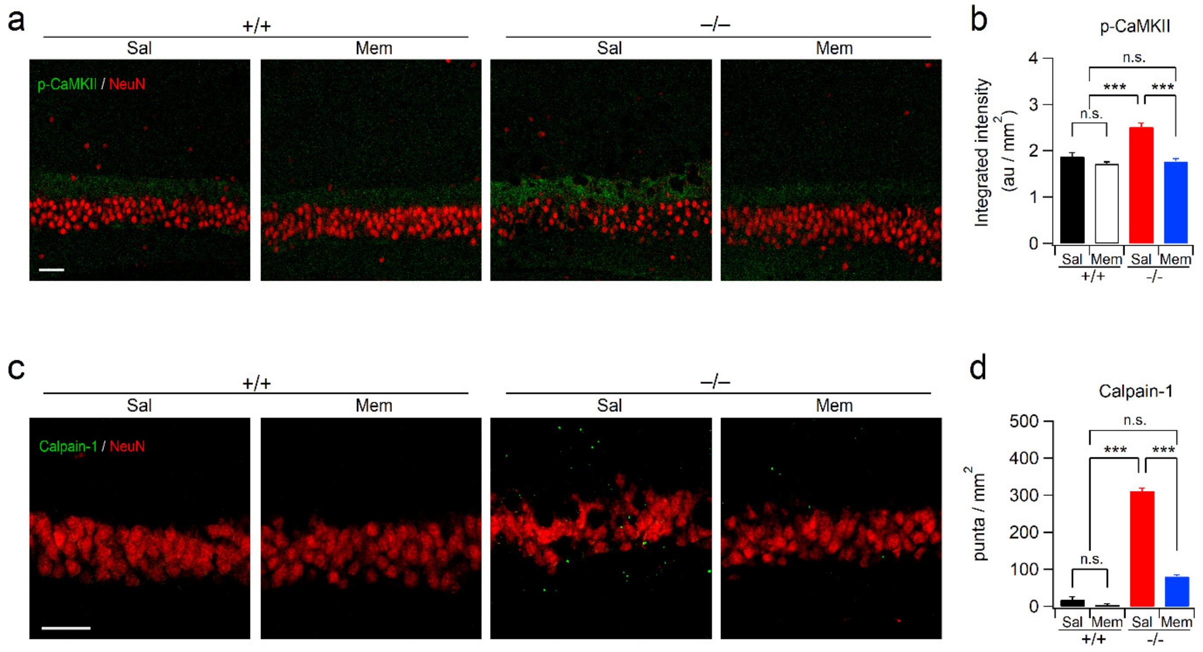

Supplementary Fig. 4. Chronic memantine treatment suppresses NMDAR downstream signaling in the Xpnpep1$^{-/-}$ hippocampus. (a, c) Immunoreactivities of phosphorylated CaMKII (p-CaMKII; a) and Calpain-1 (c) in the Xpnpep1$^{-/-}$ CA3 region were markedly reduced by chronic memantine treatment. Hippocampal CA3 neurons were visualized with the neuronal marker NeuN (red). Scale bars, 50 μm. (b, d) Quantification of immunoreactivities for p-CaMKII (b) and Calpain-1 (d) in the CA3 regions. (b) n = 4 (+/+, Sal), 6 (+/+, Mem), 5 (−/−, Sal), 6 (−/−, Mem) sections from 3 mice for each group. Genotype, $F(1, 17) = 25.21$, $p < 0.001$; treatment, $F(1, 17) = 41.64$, $p < 0.001$; interaction, $F(1, 17) = 17.65$, $p < 0.001$. (d) n = 6 (+/+, Sal), 3 (+/+, Mem), 6 (−/−, Sal), 3 (−/−, Mem) sections from 3 mice for each group. Genotype, $F(1, 14) = 65.67$, $p < 0.001$; treatment, $F(1, 14) = 41.64$, $p = 0.0011$; interaction, $F(1, 14) = 17.65$, $p = 0.0038$. ***$p < 0.001$; n.s., not significant ($p \geq 0.05$); two-way ANOVA with the Tukey multiple comparison test.
